# Day-surgery inguinal hernia repair in the elderly: single centre experience

**DOI:** 10.1186/1471-2482-13-S2-S28

**Published:** 2013-10-08

**Authors:** Bruno Amato, Rita Compagna, Francesca Fappiano, Roberto Rossi, Tommaso Bianco, Michele Danzi, Antonello Accurso, Raffaele Serra, Giovanni Aprea, Salvatore Massa

**Affiliations:** 1 Department of Clinical Medecine and Surgery University of Naples Federico II Via S. Pansini, 5 - 801311 Napoli, Italy; 2 Department of Medical and Surgical Science -University Magna Gracia of Catanzaro - Viale Europa, Località Germaneto - 88100 Catanzaro, Italy

## Abstract

**Background:**

Inguinal hernioplasty is well established as a day-surgery procedure, our purpose is to assess the safeness of this approach in elderly patients.

**Methods:**

A total of 292 inguinal hernioplasty were performed between June 2009 and February 2013. Patients were divided into 3 groups depending on the age and postoperative complications were compared in these groups.

**Results:**

Despite of a large number of higher risk (ASA 3-4) patients and a higher rate of comorbidity in older patients, unplanned admission postoperative, symptoms and complications were comparable with those for the younger patients.

**Conclusions:**

Ambulatory surgery is feasible also in older patients. Age, comorbidity and higher ASA risk should not be a barrier to elective day surgery.

## Background

Inguinal hernia repair is one of the commonly surgical procedure with good results and minimal morbidity and it is usually performed in an ambulatory surgery setting unless coexisting medical conditions merit hospitalization for specialized monitoring or care[1-3]. Inguinal hernia is more frequent in elderly than in younger patients because of loss of strength of the abdominal wall and conditions which increase intraabdominal pressure [4,10,11]. Also the ageing population is increasing, with a corresponding increasing demand for surgical services [5]. For this reason there is a growing interest in the use of this procedure in elderly patients and recent studies suggest that it is quite safe [7-9] despite the previous idea that is better a conservative approach [5,6]. In our study we evaluate the impact of day surgery approach in octogenarians.

## Methods

In the study we included patients with inguinal hernia that reached our department in the period between June 2009 and February 2013. Selection criteria used were: absence of unstable medical condition, availability of a carer overnight, access to a telephone, BMI < 32. Both unilateral and bilateral hernias were included, recurrent and complicated hernias were excluded. A total of 292 surgeries were performed by resident surgeons. Patients were divided into three age classes: a) under 75; b) between 75 and 85; c) over 85. All procedures were performed as described by Rutkow and Robbins [12] or with Lichtenstein techniques with standard-weight polypropylene mesh [13]. Prophylactic antibiotics were not used [14]. American Society of Anaesthesiologists (ASA) grade 1 and 2 patients underwent deep sedation (midazolam, fentanyl) combined with a field block of local anaesthesia (lidocaine without adrenaline) and were monitored by pulse oximetry. For ASA grade 3 and 4 patients procedures were performed in the presence of the anaesthetist and deep sedation was not used [8,15-18]. Patients were discharged within 2 h of completion of the operation after a clinical control with detailed postoperative instructions, analgesics and a 24 h contact number. Patients were telephoned by a nurse at 24\48 h postoperatively and they were asked informations about swelling, haematoma, possible wound infections. Follow up were made after 1 week, and second time after 1 month.

## Results

Comorbidity found are described in table 1.

**Table 1:** Patient Characteristics

N = 292			
**Age**	**Under 75 121 (41.4%)**	**75 - 85 133 (45.5%)**	**Over 85 38 (13%)**

ASA 1-2	90 (30.8%)	73 (25%)	9 (3%)
ASA 3-4	31 (10.6%)	60 (20.5%)	29 (10%)
Hypertension	110 (90%)	125 (94%)	37 (97%)
CD	85 (70.2%)	105 (79%)	36 (95%)
Diabetes	73 (60.3%)	40 (30%)	4 (10.5%)
Renal Failure	2 (1.6%)	3 (2.2%)	2 (5.2%)
COPD	18 (14.9%)	27 (20.3%)	11 (29%)

Although most of the patients in both groups were successfully discharged, a small proportion required overnight admission caused by a variety of complications and the number of unplanned admission was lightly higher in older patients (Table 2) but overall not particularly high. Main complications occurred were recurrence, wound infections (most in over 85 patients), wound hematoma. There were no deaths or major complications and all admitted patients were discharged the next day. Occurrence of unplanned contact with healthcare services was low and not significantly different between the three groups: 7 patients (5,7%) in the under 75 group; 6 patients (4,5%) in the 75/85 group; 2 patients (5,2%) in the over 85 group. 3 The main reasons for calling the healthcare service were nausea, vomiting and pain. Sometimes patients only needed a psychological comfort.

**Table 2:** Patient Complications

N = 292			
**Age**	**Under 75 121 (41.4%)**	**75 - 85 133 (45.5%)**	**Over 85 38 (13%)**

Recurrence	4 (3.3%)	9 (6.7%)	1 (2.6%)
Wound infection	1 (0.8%)	5 (3.7%)	2 (5.2%)
Wound hematoma	1 (0.8%)	2 (1.5%)	1 (2.6%)
Nerve entrapment	3 (2.5%)	2 (1.5%)	1 (2.6%)
Mesh infection	2 (1.6%)	3(2.2%)	1 (2.6%)
Severe bleeding	1 (0.8%)	3(2.2%)	1 (2.6%)
Arrhythmia	0	1 (0.75%)	2 (5.2%)

## Conclusions

Data collected in this study show that hernia repair can be safely offered to elderly patients with no significant increase in complications and unplanned admissions compared with younger patient, also in over 85 patients, despite the higher percentage of comorbidity found. We also note that the telephone healthcare service helped to reduce the anxiety and ensure the patient safety. Whereas day surgery has many advantages, including avoidance of stress, reduction of the risk of contracting nosocomial infection and cost savings for the hospital, we conclude that age, comorbidity and higher ASA risk should not be a barrier to elective day surgery for inguinal hernioplasty.

## List of Abbreviations

ASA: American Society of Anaesthesiologists

COPD: chronic obstructive pulmonary disease

BPH: benign prostatic hypertrophy

CD: cardiovascular disease

BMI: body mass index

## Competing interests

The authors declare that they have no competing interests.

## Authors' contributions

BA: conception and design, interpretation of data, given final approval of the version to be published.

RC, FF, RR, TB, MD, AA, RS, GA: acquisition of data, drafting the manuscript, given final approval of the version to be published

SM: conception and design, given final approval of the version to be published

## Authors' information

BA: Associate Professor of Surgery at University "Federico II" of Naples, Italy.

RC: Post-Graduate Doctorate in Vascular Surgery at University "Federico II" of Naples.

FF, RR, TB: Resident in General Surgery Training Programme at University "Federico II" of Naples.

MD, AA, GA: Aggregate Professor of Surgery at University "Federico II" of Naples, Italy.

RS: Assistant Professor of Surgery at University Magna Graecia of Catanzaro,

SM: Full Professor of Surgery at University "Federico II" of Naples, Italy.

## Declarations

Funding of this supplement has come from University funds.

This article has been published as part of *BMC Surgery *Volume 13 Supplement 2, 2013: Proceedings from the 26th National Congress of the Italian Society of Geriatric Surgery. The full contents of the supplement are available online at http://www.biomedcentral.com/bmcsurg/supplements/13/S2
